# Comparison of Unnoticed Glove Perforations during Minimally Invasive versus Open Surgeries: A Systematic Review and Meta-Analysis

**DOI:** 10.3390/children9020179

**Published:** 2022-02-01

**Authors:** Sachit Anand, Zenon Pogorelić, Apoorv Singh, Carlos Martin Llorente Muñoz, Nellai Krishnan, Anjan Kumar Dhua, Prabudh Goel, Minu Bajpai

**Affiliations:** 1 Department of Pediatric Surgery, Kokilaben Dhirubhai Ambani Hospital, Mumbai 400053, India; 2 Department of Pediatric Surgery, University Hospital of Split, 21000 Split, Croatia; zpogorelic@gmail.com; 3 Department of Surgery, School of Medicine, University of Split, 21000 Split, Croatia; 4 Department of Pediatric Surgery, All India Institute of Medical Sciences, New Delhi 110029, India; dr.singhapoorv@gmail.com (A.S.); nellai93@gmail.com (N.K.); anjandhua@hotmail.com (A.K.D.); prabudh.aiims@gmail.com (P.G.); bajpai2b@gmail.com (M.B.); 5 Surgical Clinic Medix-Muñoz, 28000 Madrid, Spain; llorentecm@gmail.com

**Keywords:** surgical gloves, glove breakage, glove puncture, glove perforation, personal protective equipment, healthcare-associated infection, minimally invasive surgery, laparoscopy

## Abstract

Objective: Various studies have depicted the incidence of glove perforations during open (OS) and minimally invasive surgeries (MIS). The aim of this meta-analysis was to compare the incidence of macroscopic and microscopic glove perforations during MIS and OS. Methods: The review was conducted in accordance with the Preferred Reporting Items for Systematic Reviews and Meta-Analyses (PRISMA) guidelines. Scientific databases (PubMed, Web of Science, Scopus, and EMBASE) were systematically searched for comparative studies depicting the glove perforation rates during MIS and OS. Risk ratios (RR) were calculated for both the outcomes (dichotomous) and the Mantel–Haenszel method was utilized for the estimation of pooled RR. The methodological quality assessment was performed by two independent investigators using the Downs and Black scale. The main outcomes of the study were the proportion of gloves with gross (macroscopic) perforations and the proportion of gloves with microscopic perforations. Results: Four comparative studies including a total of 1428 gloves (435 from the MIS group) were included. Pooling the data demonstrated no difference in the incidence of macroscopic glove perforations among the MIS and OS groups (RR 0.57, 95% CI 0.21 to 1.54, *p* = 0.27). On the other hand, the incidence of microscopic perforations was significantly higher in the OS group versus the MIS group (RR 0.72, 95% CI 0.55 to 0.95, *p* = 0.02). However, all the studies had a moderate risk of bias. Conclusions: When compared to OS, the macroscopic glove perforation rate during MIS showed no significant difference. The incidence of microscopic glove perforations was significantly higher during OS as compared to MIS. However, due to the moderate risk of bias of the available comparative studies, the level of evidence of these studies is limited.

## 1. Introduction

Appropriate usage of surgical gloves is pivotal for the prevention of transmission of infections from the patient to the surgeon and vice versa. Irrespective of the material of the glove used, perforations frequently occur in clinical settings. It has been documented that the glove perforation rate during surgeries can be as high as 30%, with the majority of these perforations occurring during major surgical procedures [1,2,3]. Of these, up to 70% of the perforations can go undetected during the entire surgery [4]. This increases the risk of the transmission of infections such as Human Immunodeficiency Virus (HIV), Hepatitis C Virus (HCV), and Hepatitis B Virus (HBV) [5]. These perforations also tend to increase the incidence of surgical site infections (SSI) in the patients [6,7].

The literature describes various methods to decrease the incidence of glove perforations such as double gloving, frequent glove change, etc.; however, the usefulness of these measures remains doubtful [8,9,10]. Various studies have compared and commented upon the incidence of glove perforations during minimally invasive (MIS) versus open surgeries (OS). Laine et al. [11] had depicted a higher incidence of glove perforations during the open versus the laparoscopic approach of abdominal surgery. Similarly, compared to the conventional thoracotomy, a significant reduction in the incidence of glove perforations was observed with the use of the thoracoscopic approach by Kojima et al. [12]. In contrast, few other studies demonstrate no significant difference in the incidence of glove perforations between the OS and MIS [13]. Moreover, Walczak et al. [14] had noticed a higher incidence of glove perforations during laparoscopic versus open cholecystectomy. Therefore, a consensus statement regarding this subject is lacking.

The present study aimed to compare the incidence of glove perforations in MIS versus OS. We also intend to systematically summarize the available literature and outline various factors that may influence the incidence of glove perforations.

## 2. Materials and Methods

### 2.1. Search Process

This review was conducted in accordance with the Preferred Reporting Items for Systematic Reviews and Meta-Analyses (PRISMA) guidelines [15]. The present review was not applicable for registration in a prospective register (e.g., PROSPERO) as we had commenced the data extraction prior to registration. To identify the already published literature and to confirm the absence of meta-analyses on this subject, two authors (SA and ZP) performed a preliminary search in the PubMed database on 14 August 2021. Subsequently, a systematic literature search was conducted by both authors on the same day. Four databases, including PubMed, Web of Science, Scopus, and EMBASE were explored using the following search keywords: (glove puncture OR glove perforation OR glove breakage OR glove tear) AND (minimally invasive surgery OR laparoscopic surgery OR thoracoscopic surgery). The search process is briefly highlighted in Appendix A. The duplicate records were removed from the search results and the remaining studies were screened for eligibility.

### 2.2. Eligibility

The inclusion criteria used were: *Participants*—all the gloves that were worn by the surgical team during minimally invasive surgeries (MIS); *Intervention*—identification of surgical glove perforation(s) after the procedure; *Comparison*—the gloves that were worn by the surgical team during open surgeries (OS); *Outcomes*—the proportion of gloves with gross (macroscopic) perforations and the proportion of gloves with microscopic perforations were the main outcomes considered. In addition, the factors influencing the rates of glove perforation were also identified and discussed descriptively.

All the studies reporting one of the main outcomes were included in this review. In addition, an attempt was made to include only those studies that have utilized established methods for detection of glove perforations, i.e., water leak test (WLT) and electrical resistance test (ERT) for macroscopic and microscopic perforations, respectively [14]. All the comparative studies reporting glove perforations were eligible for inclusion, and neither the type of surgery nor the body compartment that was operated upon was considered as specific eligibility criteria. The studies where gloves were changed due to conspicuous perforations before or during the surgery were excluded. Case reports, editorials, letters to the editors, opinion articles, and conference abstracts were also excluded. In addition, studies with unavailable full texts were excluded.

### 2.3. Data Extraction

Two investigators (AD and PG) independently performed the data synthesis in Microsoft Excel (Version 15.24) spreadsheets. Any disagreements among them were resolved via discussion with another investigator (MB). Apart from the outcome data, the information regarding the name of the author, year of publication, type of study design, number of gloves assessed in each study, and the number of gloves in each treatment group was extracted.

### 2.4. Quality Assessment

Two investigators (AS and NK) independently assessed the quality of the included studies. The validated Downs and Black scale was utilized for the quality assessment [16]. This twenty-seven-item questionnaire has four domains with total scores ranging from 0 to 32. On the basis of the total scores assigned to each study, the risk of bias was graded as high (score = 0–15), moderate (score = 16–23), or low (score > 23). The inter-observer agreement regarding the scoring of each item of each included study was declared using the kappa statistics [17]. On the basis of the power of kappa, the degree of agreement was defined as almost perfect (0.81–1.00), substantial (0.61–0.80), moderate (0.41–0.60), fair (0.21–0.40), and slight (0.00–0.20).

### 2.5. Statistical Analysis

The baseline data were expressed as numbers, proportions, averages, and ranges. The meta-analysis was performed using RevMan 5.4 (Cochrane Collaboration, London, UK). For both the outcomes (dichotomous), the risk ratios (RR) with 95% confidence intervals (CI) were estimated. The Mantel–Haenszel method was utilized for the calculation of pooled risk ratio [18]. The level of heterogeneity among the included studies was evaluated using the I^2^ statistics. A random-effects model was used in case of substantial heterogeneity (I^2^ > 50%). A *p*-value of <0.05 was considered statistically significant.

## 3. Results

### 3.1. Baseline Data

**Figure 1:**
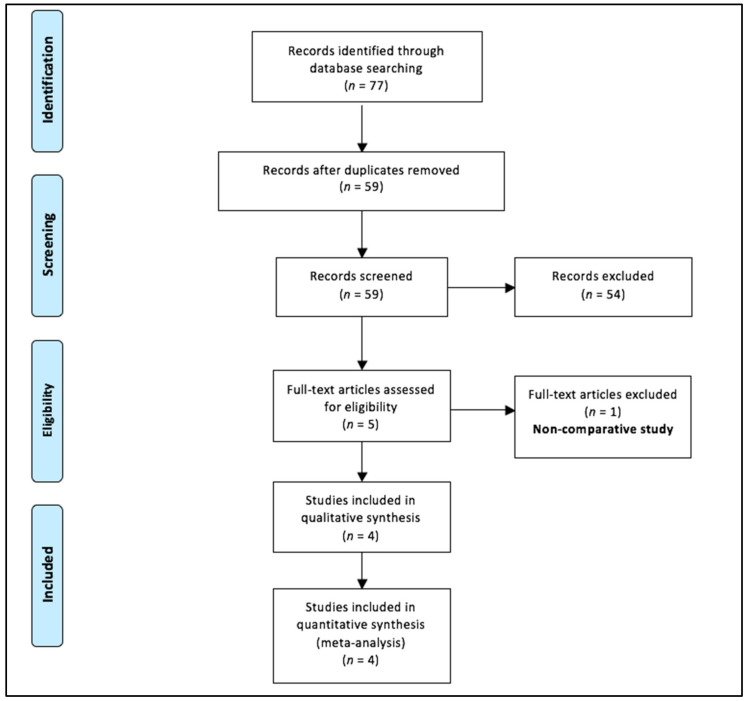
Selection of the relevant studies using the Preferred Reporting Items for Systematic Review and Meta-Analysis (PRISMA) flow diagram.

Out of seventy-seven records identified with our search strategy, eighteen duplicate articles were removed. The remaining fifty-nine articles were screened for eligibility. Of these, fifty-four abstracts were excluded and only five full texts were assessed for inclusion (Figure 1). One of them was a non-comparative study and was further excluded [19]. Therefore, only four studies were included in the final meta-analysis [11,12,13,14]. The study designs of these studies were cross-sectional (*n* = 2), prospective study (*n* = 1), and randomized controlled trial (*n* = 1). One study each demonstrated the glove perforation rates following open versus minimally invasive urological [13], thoracic [12], and abdominal [11] procedures. The remaining study compared the incidence of glove perforations after laparoscopic versus open cholecystectomy [14].

The baseline characteristics of the included studies are demonstrated in Table 1. A total of 1428 gloves were included in this meta-analysis. Of these, 435 and 993 gloves belonged to the MIS and OS groups, respectively. Different types of gloves were used by the surgeons in the included studies, e.g., latex-based, latex-free polyisoprene-based, double indicator gloves, etc. (Table 1). The methods used to detect the perforations were standard. The macroscopic and microscopic perforations were detected by the WLT and ERT, respectively.

### 3.2. Summary of the Included Studies

#### 3.2.1. Laine et al., 2004

This randomized study from Finland compared the macroscopic glove perforation rate after laparoscopic versus open abdominal surgeries. The surgeons were randomized to use either of the three glove brands (one type was double indicator gloves). Perforations were more common in open surgeries (9.6% versus 3.3%). The frequency of single versus double glove perforations did not differ within the MIS and OS groups. However, compared to the double indicator gloves, a significantly higher number of perforations were identified in the single gloves in both the treatment groups. Perforations were more common in the index finger of the non-dominant hand in the gloves worn by primary surgeons, and in surgeries lasting for >2 h.

#### 3.2.2. Kojima et al., 2005

This prospective study from Japan compared the unnoticed glove perforations following thoracoscopic versus open thoracic surgeries. The procedure perforation rate (25% and 70% in the MIS and OS groups, respectively) and the glove perforation rate (12% and 41% in the MIS and OS groups, respectively) were significantly lower following the thoracoscopic surgeries. A longer duration of surgery (>2 h) was associated with a higher glove perforation rate in the MIS group. However, there was no relationship between operative duration and glove perforation rate among the gloves retrieved from the OS group.

#### 3.2.3. Feng et al., 2011

This cross-sectional study from the United States of America demonstrated the macroscopic and microscopic glove perforation rates following urologic procedures. A total of 180 gloves from two different brands were tested for perforations. Out of these, 59 gloves were used in endourology procedures. Therefore, these gloves were excluded and a total of 121 gloves were included in the present meta-analysis. Gross perforations were appreciated in only five and six gloves from the MIS and OS groups, respectively. Microscopic perforations were more common among the OS group. In this study, no association was observed between the glove perforation rate and the duration of surgery. In addition, none of the other variables, including glove size, glove brand, glove handedness (left or right), and role in surgery (primary surgeon or assistant) had an influence on the glove perforation rate.

#### 3.2.4. Walczak et al., 2015

This cross-sectional study was conducted in Poland. Both macroscopic and microscopic perforations were detected in the gloves worn by the primary surgeon and the first assistant during laparoscopic or open cholecystectomy. A total of 376 gloves, 192 and 184 belonging to the MIS and OS groups, were screened for any perforations. Interestingly, the overall perforation rate was significantly higher in the gloves retrieved from the MIS versus the OS groups. The macroscopic perforations were more common after the MIS, while microscopic perforations were more common after the OS. Gloves worn by the primary surgeons and on the non-dominant hand were more likely to perforate.

### 3.3. Methodological Quality Assessment

The quality assessment of the included studies by the Downs and Black scale is demonstrated in Table 2. All the included studies had a moderate risk of bias. The average scores assigned to the studies ranged from 17.5 to 20. The minimum and maximum scores were assigned to the studies by Kojima et al. [12] and Feng et al. [13], respectively. The inter-observer agreement was almost perfect (Kappa = 0.939, *p* < 0.0001).

### 3.4. Meta-Analysis

#### 3.4.1. Macroscopic Glove Perforations

**Figure 2:**
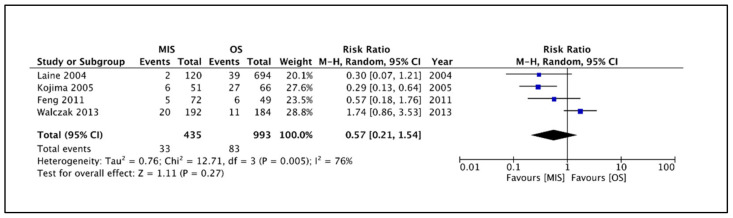
Forest plot comparison between the two patient groups in terms of the incidence of macroscopic glove perforations. Legends: MIS, minimally invasive surgery group. OS, open surgery group. M-H, Mantel–Haenszel method. CI, confidence interval.

This outcome was reported by all four included studies [11,12,13,14]. Macroscopic glove perforations were detected in 435 and 993 gloves retrieved from MIS and OS groups, respectively. A total of 116 perforated gloves were identified. Of these, 33 and 83 belonged to the MIS and OS groups. Pooling the data (Figure 2) showed no significant difference in the rates of macroscopic perforations among the two groups (RR 0.57, 95% CI 0.21 to 1.54, *p* = 0.27). The estimated heterogeneity among the included studies was substantial and statistically significant (I^2^ = 76%, *p* = 0.005) for this outcome.

#### 3.4.2. Microscopic Glove Perforations

**Figure 3:**
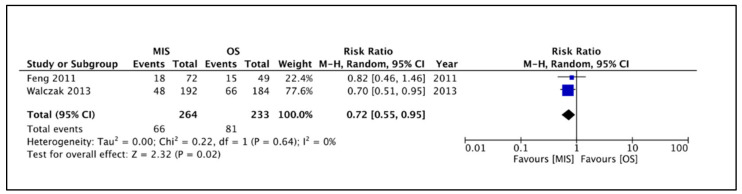
Forest plot comparison between the two patient groups in terms of the incidence of microscopic glove perforations. Legends: MIS, minimally invasive surgery group. OS, open surgery group. M-H, Mantel–Haenszel method. CI, confidence interval.

This outcome was reported by only two studies [13,14]. The rates of microscopic perforations were compared among 264 and 233 gloves retrieved from the MIS and OS groups, respectively. A total of 147 microscopic perforations, 66 and 81 belonging to the MIS and OS groups, respectively, were detected in these gloves. The pooled risk ratio (Figure 3) for the occurrence of microscopic perforations in gloves belonging to the MIS group versus the OS group was 0.72 (95% CI 0.55 to 0.95), demonstrating a statistically significant difference (*p* = 0.02). For this outcome, the heterogeneity was neither substantial nor statistically significant.

#### 3.4.3. Factors Influencing the Glove Perforation Rates in the Included Studies

*Duration of surgery*: The duration of surgery was discussed in three included studies [11,12,13]. Two studies reported a higher incidence of glove perforation following prolonged surgeries, i.e., more than 2 h [11,13]. However, the difference was not statistically significant. The study by Kojima et al. [12] highlighted a significantly higher incidence of glove perforations among the thoracoscopic surgeries lasting for >2 h. However, in the same study, the duration of surgery had no influence on those operated via the conventional open approach.

*Role in surgery*: Three studies assessed and compared the glove perforation rates among the primary surgeons and assistant surgeons [11,13,14]. Two of them reported a significantly higher incidence of perforations in the gloves worn by the primary surgeon [11,14]. However, the third study by Feng et al. [13] revealed no significant difference in the glove perforation rates among the primary surgeons versus the assistants.

*Glove handedness*: While two studies [12,13] showed no difference in the perforation rates among the gloves worn in a particular hand, the non-dominant hand glove was more prone to perforate in the remaining two studies [11,14]. Also, the portion of the glove over the index finger was more susceptible for perforation as compared to the rest of the glove.

*Number of gloves*: Only one study [11] demonstrated this variable. When perforation rates were compared among the single versus double indicator gloves, a significantly lower rate of unnoticed perforations was identified among the latter. This observation was common between both the MIS and OS groups.

## 4. Discussion

Originally designed as a gear to protect healthcare workers from caustic disinfectants, surgical gloves are an essential adjunct in minimizing surgical site infections in patients and prevents the transmission of infections such as HIV, HCV, and HBV. Studies have estimated the risk of a surgeon developing HIV infection due to damaged gloves as 1 in 1500 [5]. The most used surgical gloves are natural latex gloves. When these gloves are exposed to fluids, the space between the rubber particles (containing proteins, fatty acids, and salts) gets partially dissolved, resulting in the formation of channels by a process known as hydration. This increases the conductance of the gloves and subsequently increases the risk of electrical injury to the surgeon. Moreover, this process can make the glove more susceptible to punctures and tears [20,21,22].

Several studies have shown that the incidence of macroscopic glove perforations in OS is higher than MIS, with some studies reporting the perforation rate as high as 30% in OS [1]. However, the current analysis shows that there is no significant difference in the macroscopic perforation rate between the two groups. This could be attributed to the fact that although in open surgeries there is a higher degree of mechanical trauma to the gloves (specifically in thoracic and orthopedic surgeries); however, in MIS, the concentration of the surgeon is more towards the screen and hence there is an increased risk of glove injury while managing the extracorporeal portion of the long MIS instruments [12]. An interesting observation in this regard was made by Walczak et al. [14]. They had noticed that the most common point of glove perforation in laparoscopic surgeries was while suturing of the port sites in the abdominal wall at the end of the surgery, indicating that fatigue towards the end of the procedure may also be a contributory factor.

Only two of the included studies in the present meta-analysis commented on the incidence of microscopic perforations, and the results indicate that they are higher in the OS group. This can be easily explained by the fact that in the OS group, the operator’s and assistant’s gloves come in direct contact with electrosurgical instruments, needles, and blades that have sharp edges or tips. On the contrary, the laparoscopic instruments have sharp edges or pointed tips far away from the instrument’s grips. In addition, in MIS, there is significantly less manipulation and change of the instruments during the surgery. Frequent changes of instruments during the open surgery may also cause micro-damage to the gloves. In addition, during the thoracic and orthopedic surgeries via open approach, the commonly encountered sharp bony spicules can invariably lead to higher chances of glove penetration and perforations [12].

A variety of factors influence the rate of glove perforations in both minimally invasive as well as open surgeries. Of these, the duration of the surgery is the most widely discussed variable [23]. As reported, various studies have documented a higher incidence of glove perforations with an increased duration of surgery [11,12,13]. Similarly, few studies have also explored the impact of other factors, e.g., the role of the surgeon in surgery, handedness of the surgeon, the number of gloves, etc. on the glove perforation rate [11,12,13,14,19,24]. Although these factors do seem to affect the perforation rate, the current published literature is not sufficient to draw a significant consensus in this regard.

The existing literature has also highlighted that glove perforations increase the transmission of pathogens. This was demonstrated in urologic procedures by Hübner et al. [25], with the data showing that micro-perforations allow passage of bacteria in up to 54% of cases. However, with respect to the perforations leading to SSI in the patients, a recent study by Matsuoka et al. [18] showed that a clear correlation could not be drawn between glove perforation and SSI. The cultures obtained from most of the SSI sites in their colorectal surgery patients had shown growth of enterobacteria rather than skin flora. Nevertheless, the microbe transmission from the surgeon to the patient can be easily prevented by double gloving [26,27]. Contrary to the common belief that double gloving decreases the tactile sensation, the study by Fry et al. [28] had shown that this is not the case. Therefore, double gloving has no potential demerits and needs to be encouraged in both MIS and OS. Additionally, several studies also recommend changing the gloves after 90 to 150 min from the commencement of surgery to decrease the risk of glove perforations [29,30,31].

Previous studies on glove perforations during pediatric surgeries have demonstrated that the incidence of glove rupture during these procedures is around 10–15% [32,33]. In pediatric orthopedic surgeries, Al-Habdan et al. [34] have highlighted the superiority of double gloves versus single gloves. The authors have also mentioned that in the absence of double gloves, single gloves need to be changed every hour to prevent fluid contact between patients and surgeons. 

The results of this meta-analysis must be interpreted within the context of a few limitations. First, all the included studies had a moderate risk of bias. Second, the sample size of these studies was also limited. Third, a non-uniform reporting of the incidence of microscopic perforations was observed among the included studies. Only two studies had reported this outcome variable. Furthermore, the reporting of the factors influencing the glove perforation rate was also highly variable. Fourth, important information on the incidence of SSI due to glove perforations was not present in these included studies. Finally, this meta-analysis depicted the glove perforation rates of different surgeons operating in different body compartments/organs. Outcome differences can also arise due to differences in their operative experience. The composition (make) of gloves was also different in these studies. Therefore, further studies need to be conducted to address these important factors before any definite conclusions are drawn.

Despite the above limitations, the present meta-analysis is the first to compare the glove perforation rates during MIS and OS. As per the available comparative studies, there is no difference in the macroscopic glove perforation rates during MIS and OS. On the other hand, the microscopic glove perforation rate was significantly higher during OS as compared to MIS. However, due to the moderate risk of bias of the included studies, an appropriate estimate of the overall effect is difficult to derive. The strengths of the present review include reporting and external validity, while the weaknesses lie in internal validity and power.

## 5. Conclusions

When compared to OS, the macroscopic glove perforation rate during MIS showed no significant difference. The incidence of microscopic glove perforations was significantly higher during OS as compared to MIS. However, due to the moderate risk of bias of the available comparative studies, an appropriate estimate of the overall effect cannot be derived. Therefore, well-designed randomized controlled trials need to be conducted before any definite conclusions are drawn.

## Figures and Tables

**Table 1:** Baseline characteristics of the included studies.

Author	Study Design	Sample Size (N of Gloves) MIS OS		Body Compartment/ Organ/Organ System Operated	Composition of Gloves	Tests for Identification of Perforation
Laineet al., 2004 [11]	RCT ^§^	120	694	Abdominal (elective and emergency) surgeries	Single (latex or latex-free) or double indicator gloves ^#^	WLT
Kojimaet al., 2005 [12]	Pro	51	66	Thoracic operations	Latex-based gloves	WLT
* Feng et al., 2011 [13]	Cross	72	49	Urologic surgeries ^†^	Latex-free PI made gloves (2 different brands)	Both WLT and ERT
* Walczak et al., 2013 [14]	Cross	192	184	Cholecystectomy	Three brands of latex, powdered gloves	Both WLT and ERT

* These studies reported the occurrence of microscopic glove perforations. On the other hand, macroscopic perforations were depicted in all four studies. ^§^ The surgeons were randomized to utilize either single or double indicator gloves. ^†^ Apart from laparoscopic and open, endoscopic cases were also studied. However, those were not included in this meta-analysis. ^#^ Three different brands were used according to surgeon’s preference.

**Table 2:** Downs and Black scale scores for the included studies by author 1 and author 2. The total scores and inter-observer agreement are also depicted in the table.

Study	Reporting	External Validity	Internal Validity-Bias	Internal Validity-Confounding	Power	Total Scores
*Methodological assessment by author 1*						
Laine et al. 2004 [11]	9	3	5	3	0	20
Kojima et al. 2005 [12]	7	3	4	3	0	17
Feng et al. 2011 [13]	9	3	5	3	0	20
Walczak et al. 2015 [14]	8	3	5	3	0	19
*Methodological assessment by author 2*						
Laine et al. 2004 [11]	8	3	5	3	0	19
Kojima et al. 2005 [12]	8	3	4	3	0	18
Feng et al. 2011 [13]	9	3	5	3	0	20
Walczak et al. 2015 [14]	9	3	5	3	0	20
*Total scores and inter-observer agreement*						
**Study**	**Rater 1**	**Rater 2**	**Mean**	**Kappa Value**	***p*-Value**	
Laine et al. 2004 [11]	20	19	19.5	0.939	<0.0001	
Kojima et al. 2005 [12]	17	18	17.5			
Feng et al. 2011 [13]	20	20	20			
Walczak et al. 2015 [14]	19	20	19.5			

## Data Availability

The data presented in this study are available upon request of the respective author.

## Abbreviations

Cross	cross-sectional study.
Pro	prospective cohort.
RCT	randomized controlled trial.
MIS	minimally invasive surgery group.
OS	open surgery group.
PI	polyisoprene.
WLT	water leak test.
ERT	electrical resistance test,

## Appendix A

**PubMed**—((((glove puncture) OR (glove perforation)) OR (glove breakage)) OR (glove tear)) AND (((minimally invasive surgery) OR (laparoscopic surgery)) OR (thoracoscopic surgery))

**Web of Science**—Query 1: ((ALL = (glove puncture)) OR (glove perforation)) OR (glove breakage)) OR (glove tear) AND Query 2: ((ALL = (minimally invasive surgery)) OR (laparoscopic surgery)) OR (thoracoscopic surgery)

**Scopus**—(TITLE-ABS-KEY (“glove puncture” OR “glove perforation” OR “glove breakage” OR “glove tear”) AND TITLE-ABS-KEY (“minimally invasive surgery” OR “laparoscopic surgery” OR “thoracoscopic surgery”)

**EMBASE**—(‘glove puncture’: ti,ab,kw OR ‘glove perforation’: ti,ab,kw OR ‘glove breakage’: ti,ab,kw OR ‘glove tear’:ti,ab,kw) AND (‘minimally invasive surgery’: ti,ab,kw OR ‘laparoscopic surgery’: ti,ab,kw OR ‘thoracoscopic surgery’: ti,ab,kw)

**Database**	**Studies**
PubMed	47
Web of Science	21
Scopus	6
EMBASE	3
Total	77
Duplications	18
After duplication removal	59
